# Headache and Status Epilepticus in the Postpartum Period; Posterior Reversible Encephalopathy Syndrome or Cerebral Venous Thrombosis?

**DOI:** 10.1155/2013/680327

**Published:** 2013-04-24

**Authors:** Panagiotis Zis, Antonios Tavernarakis

**Affiliations:** Department of Neurology, Evangelismos General Hospital, 10676 Athens, Greece

## Abstract

We report a case of a young woman, with a history of a miscarriage and a molar pregnancy, who developed headache and status epilepticus in postpartum day three. Posterior reversible encephalopathy syndrome (PRES) and cerebral venous and sinus thrombosis (CVST) can present with identical clinical picture; however, the imaging findings can help the clinician to make the correct diagnosis and initiate the appropriate treatment. Both PRES and CVST are medical emergencies and fully reversible entities especially when treatment initiation is immediate.

## 1. Introduction

Acute neurological symptoms in the postpartum women could be caused by exacerbation of a preexisting neurological condition, by initial presentation of a non-pregnancy-related problem, or by new onset neurological conditions that occur uniquely or with increased frequency just after pregnancy [1].

The clinician, either the obstetrician or the neurologist, based on the patient's history and the postpartum course, should be able to identify and evaluate the presenting symptoms. The early recognition of such neurological conditions and the initiation of the relevant treatment are crucial as most of the disorders can be fully reversible. However, any delay could even lead to the patient's death.

Neurological symptoms in postpartum women can be grouped in three broad categories: headache, seizures, and focal neurological deficits. We report a case of a gravida 1 para 3 who presented initially with headache and few hours later with status epilepticus, in the postpartum period. This case was not only challenging because of the puzzling imaging findings, medical history, and neurological signs, but also as it was a fully treatable emergency situation that involved two distinct specialties: neurology and obstetrics.

## 2. Case

A 35-year-old Caucasian, with a history of a miscarriage and a molar pregnancy, was admitted and successfully delivered a healthy girl following epidural anesthesia. According to prenatal documentation, her pregnancy was normal, without signs of preeclampsia. 

Because of her previous history of the two unsuccessful pregnancies over the last three years, she had been through a full thrombophilia screening which had revealed that antithrombin III, factor V Leiden, PCGlob-FVNR, and protein C and S levels were within normal limits. However, the molecular genetic screen had showed that the patient was heterozygous for the MTHFR C677T mutation. 

**Figure 1:**
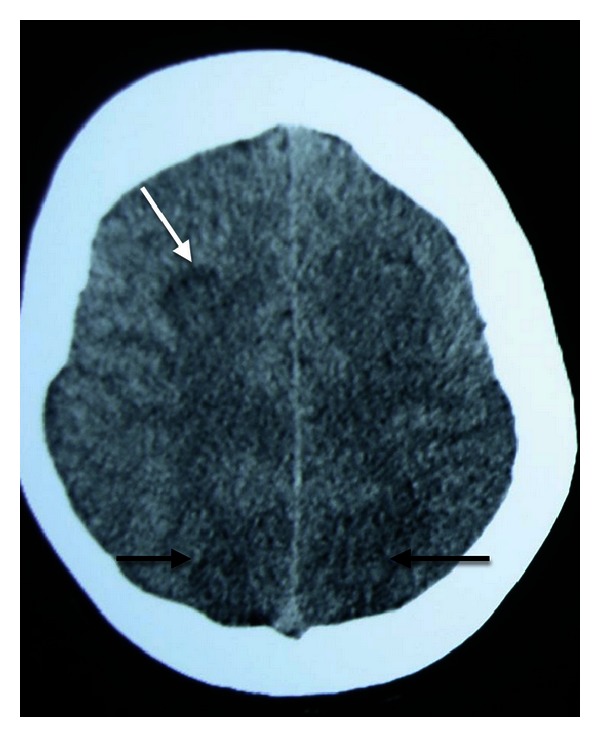
Cranial computerized tomography indicating discreet hypodensity on both occipital lobes (black arrows) and a hypodense lesion on the right parietal lobe (white arrow).

On postpartum day one, the patient complained of severe diffuse headache, dizziness, and vomiting, symptoms that were improving when lying and therefore initially were attributed to intracranial hypotension caused by the epidural anesthesia. However, on postpartum day three, the patient developed a convulsive status epilepticus for which she had to be intubated. A cranial computerized tomography (CT) showed discreet hypodensity on both occipital lobes and a hypodense lesion on the right parietal lobe (Figure 1).

Based on the history of the miscarriage and the molar pregnancy and of the fact that the patient was heterozygous for the MTHFR C677T mutation, cerebral venous thrombosis was suspected and a lumbar puncture was performed. The opening pressure of the cerebrospinal fluid (CSF) was 20 cm H_2_O. Further CSF analysis showed 11 lymphocytes per mm^3^, glucose 90 mg/dL, and total protein 128 mg/dL. All of the routine blood and urine examinations were unremarkable. 

**Figure 2:**
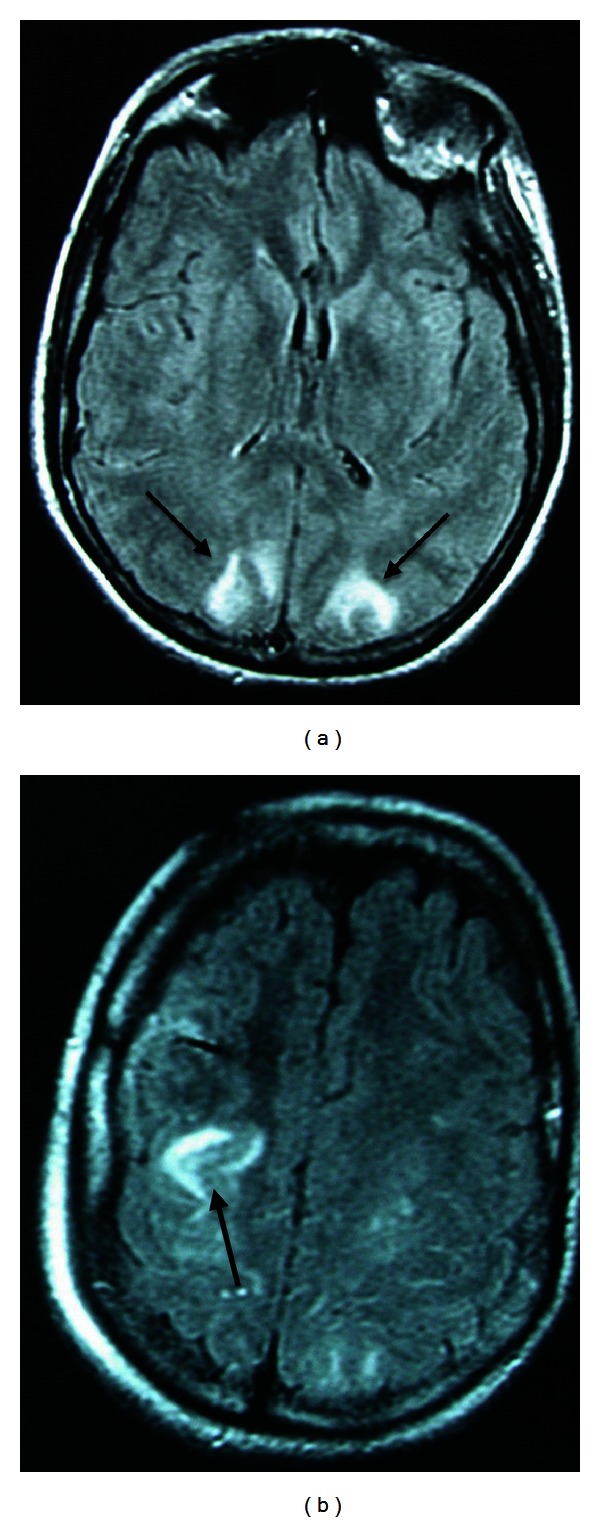
(a) Fluid attenuated inversion recovery magnetic resonance imaging indicating high signal lesions on both occipitals lobes. (b) Fluid attenuated inversion recovery magnetic resonance imaging indicating high signal lesions on the right parietal lobe.

**Figure 3:**
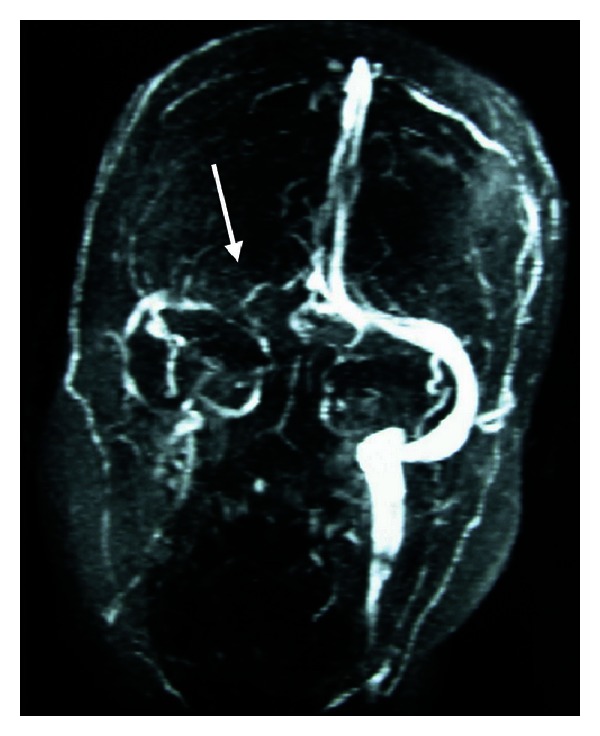
Magnetic resonance venography revealing narrowing of the right transverse sinus without the development of excessive collateral circulation.

An urgent brain magnetic resonance imaging (MRI) showed high signal lesions on both occipitals lobes and the right parietal lobe (Figure 2), when the urgent magnetic resonance venography (MRV) revealed narrowing of the right transverse sinus (Figure 3). However, because of the lack of any collateral circulation around the right transverse sinus, the narrowing of the latter was attributed to congenital hypoplasia.

**Figure 4:**
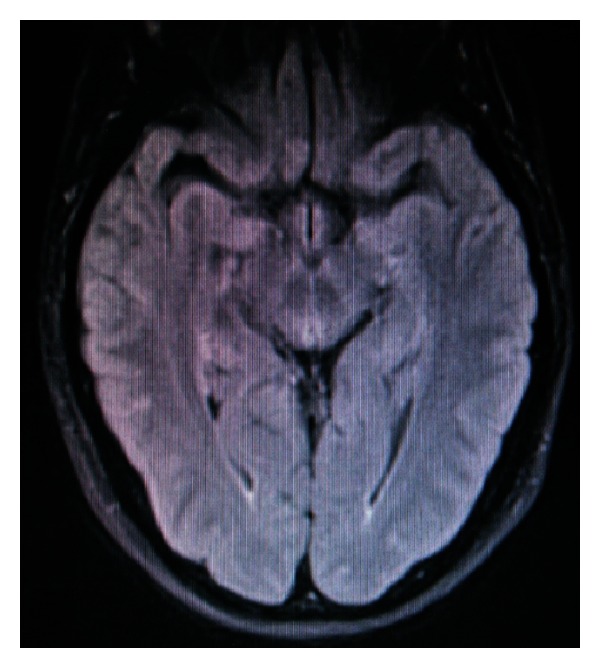
Fluid attenuated inversion recovery magnetic resonance imaging at follow up (15 days after the event) indicating that all lesions have disappeared.

The patient was extubated the next day (postpartum fourth day). Being on antiepileptic treatment, she did not suffer from any other epileptic seizures. The headache has gradually improved and the patient was discharged on postpartum day fourteen. The MRI at discharge showed that all lesions disappeared (Figure 4), and a diagnosis of posterior reversible encephalopathy syndrome was made.

## 3. Discussion

Posterior reversible encephalopathy syndrome (PRES) in the postpartum period is an infrequent diagnosis in day-to-day obstetric care [2]. The clinical manifestations of PRES are acute and self-limited, headache, altered mental status, cortical blindness, and seizures [3]. By definition, all patients with posterior reversible encephalopathy syndrome have a characteristic MRI pattern with bilateral hemispheric boundary zones of hyperintensities on T2 and FLAIR imaging, with increased apparent diffusion coefficient values, affecting the cortex and subcortical and deep white matter to varying degrees [4]. The pathogenesis of PRES remains unclear, but it appears to be related to disordered cerebral autoregulation and endothelial dysfunction [5]. PRES is increasingly recognized and reported in case reports and case series; however, its exact incidence remains not known. 

Cerebral venous and sinus thrombosis (CVST) in the postpartum period is the most common cerebrovascular incident during the puerperium [6]. Clinical manifestations consist of headache, vomiting, focal or generalized seizures, confusion, blurred vision, focal neurologic deficits, and altered level of consciousness. The headache frequently precedes other symptoms and is diffuse and often severe. CVST in some cases has been linked to thrombophilias [6, 7]. However, a recent meta-analysis showed that there is currently insufficient data supporting that MTHFR C677T mutation alone is a definite risk factor for CVST [8].

Our patient initially complained of headache, which was attributed to intracranial hypotension caused by the epidural anesthesia. However, as the headache was complicated with status epilepticus, the clinical picture could be attributed to two different neurological entities: CVST or PRES. Interestingly enough, the imaging findings were puzzling. 

The imaging findings in both the CT and the MRI could theoretically be attributed to venous infarctions, and as the MRV revealed narrowing of the right transverse sinus CVST was high in the differential diagnosis. However, the normal CSF opening pressure and the lack of extensive collateral circulation around the right transverse sinus were against CVST. Especially the latter was in favour of PRES. Moreover, the fact that the lesions were mainly in the posterior cerebral areas and eventually disappeared within 2 weeks of symptoms onset made PRES a definite diagnosis.

## 4. Conclusion

We report a case of PRES in a young woman, with a history of a miscarriage and a molar pregnancy, who developed headache and status epilepticus in postpartum day three. The clinical picture can be identical in cases of PRES and CVST; however, the imaging findings, especially in the MRI and the MRV, can help the clinician to make the correct diagnosis and initiate the appropriate treatment. Both PRES and CVST are medical emergencies and fully reversible entities especially when treatment initiation is immediate.
